# Interspecies interactions are an integral determinant of microbial community dynamics

**DOI:** 10.3389/fmicb.2015.01148

**Published:** 2015-10-20

**Authors:** Fatma A. A. Aziz, Kenshi Suzuki, Akihiro Ohtaki, Keita Sagegami, Hidetaka Hirai, Jun Seno, Naoko Mizuno, Yuma Inuzuka, Yasuhisa Saito, Yosuke Tashiro, Akira Hiraishi, Hiroyuki Futamata

**Affiliations:** ^1^Laboratory of Food Crops, Institute of Tropical Agriculture, Universiti Putra MalaysiaSerdang, Malaysia; ^2^Department of Applied Chemistry and Biochemical Engineering, Graduate School of Engineering, Shizuoka UniversityHamamatsu, Japan; ^3^Department of Environmental and Life Sciences, Toyohashi University of TechnologyToyohashi, Japan; ^4^Department of Mathematics, Shimane UniversityMatsue, Japan

**Keywords:** microbial ecosystem, population dynamics, interaction, self-organization, chemostat, phenol

## Abstract

This study investigated the factors that determine the dynamics of bacterial communities in a complex system using multidisciplinary methods. Since natural and engineered microbial ecosystems are too complex to study, six types of synthetic microbial ecosystems (SMEs) were constructed under chemostat conditions with phenol as the sole carbon and energy source. Two to four phenol-degrading, phylogenetically and physiologically different bacterial strains were used in each SME. Phylogeny was based on the nucleotide sequence of 16S rRNA genes, while physiologic traits were based on kinetic and growth parameters on phenol. Two indices, *J* parameter and “interspecies interaction,” were compared to predict which strain would become dominant in an SME. The *J* parameter was calculated from kinetic and growth parameters. On the other hand, “interspecies interaction,” a new index proposed in this study, was evaluated by measuring the specific growth activity, which was determined on the basis of relative growth of a strain with or without the supernatant prepared from other bacterial cultures. Population densities of strains used in SMEs were enumerated by real-time quantitative PCR (qPCR) targeting the gene encoding the large subunit of phenol hydroxylase and were compared to predictions made from *J* parameter and interspecies interaction calculations. In 4 of 6 SEMs tested the final dominant strain shown by real-time qPCR analyses coincided with the strain predicted by both the *J* parameter and the interspecies interaction. However, in SMEII-2 and SMEII-3 the final dominant *Variovorax* strains coincided with prediction of the interspecies interaction but not the *J* parameter. These results demonstrate that the effects of interspecies interactions within microbial communities contribute to determining the dynamics of the microbial ecosystem.

## Introduction

Microbial populations influence each other during the development of their ecosystems, while at the same time the microbial ecosystem is affected by its surrounding environments and vice versa (Fernández et al., 2000; Hashsham et al., 2000; Little et al., 2008; Klitgord and Segrè, 2010). It is predicted that the sustainability of an ecosystem will be maintained by dynamic changes of the bacterial community (dynamic equilibrium) (Ishii et al., 2012; Yamamoto et al., 2014). Comprehensive understanding of the principles of microbial ecosystems is not only central to research in microbial ecology but also important for efficient bioremediation, wastewater treatment, agriculture and human health but has been a challenging subject for microbial ecologists (Fernández et al., 1999, 2000; Futamata et al., 2005; El-Chakhtoura et al., 2015).

We previously observed a unique phenomenon of bacterial population dynamics in a chemostat soil bioreactor enriched with phenol (Futamata et al., 2005). Usually, since the chemostat culture is enriched with a low concentration of a sole substrate, bacteria exhibiting the highest affinity for this substrate eventually come to dominate the culture (Watanabe et al., 1998a). However, members of *Variovorax* exhibiting lower affinities for phenol constituted the final dominant population in this soil bioreactor (Futamata et al., 2005). This result indicated that kinetic parameters are not necessarily the sole determinant for predicting bacterial community dynamics in a chemostat culture. Therefore, further research is necessary to unveil the mechanism by which the *Variovorax* species became dominant in a complex chemostat ecosystem despite exhibiting lower affinity for phenol.

Kinetic and growth parameters have been used to predict bacterial population dynamics and the “*J* parameter” has been reported as a useful predictor for which strain will become dominant in a mixed culture (Hansen and Hubbell, 1980). Additionally, interactions between bacterial cells is also thought to be an important mechanism that plays a major role in biofilm formation through quorum sensing and other biological processes (Tashiro et al., 2013; DeSalvo et al., 2015; Inaba et al., 2015). Here, we investigated whether kinetic and growth parameters or interactions between populations of different species are more important for determining the composition of a microbial ecosystem.

The complete set of all possible interactions between many species of bacteria has to be investigated in order to fully understand a natural microbial ecosystem. However, microbial ecosystems in natural and engineered systems (including the soil bioreactor described above), are far too complex to be described by a single determinant and/or law. Currently, the factors and processes that influence the behavior and functionality of bacterial ecosystems remain largely unknown. Due to the high complexity of natural systems, an approach known as the synthetic microbial ecosystem (SME) has gained much interest of late (Narisawa et al., 2008; Mee et al., 2014; De Roy et al., 2014). Because of their reduced complexity and increased controllability, synthetic communities are often preferred over complex communities when examining ecological theories. The possible factors that influence the microbial community are reduced to a minimum, allowing the factors that affect specific community dynamics to be managed and identified.

The objective of this study was to understand the surprising bacterial community dynamics observed in the soil bioreactor. To this end, we determined the *J* parameter (based on kinetic and growth parameters) (Hansen and Hubbell, 1980) and the interspecies interactions (based on the specific growth activity) of several phenol degrading bacterial strains. These data were used to predict which strain would become dominant in different SMEs and the results were compared to real-time qPCR quantification of each strain. From this work we have determined that interactions between species, in addition to kinetic and growth parameters, are integral in determining bacterial community dynamics.

## Materials and methods

### Bacterial strains used in this study

Phenol-degrading bacteria isolated from a phenol-degrading soil bioreactor and its inoculum (Futamata et al., 2001a, 2005) were used in this study (Table 1). *Pseudomonas putida* strains P-2, P-5, P-6, and P-8, and *Ralsotonia* sp. strain P-10 were isolated from trichloroethene (TCE) -contaminated aquifer soil (Futamata et al., 2001a). Other strains were isolated from chemostat enrichment cultures grown on phenol with pristine aquifer soil sampled from near the TCE-contaminated site. These bacteria were grown at 25°C in BSM medium supplemented with phenol at 2.0 mM (Futamata et al., 2001a) or MP medium (Watanabe et al., 1998a) supplemented with phenol at 2.0 mM.

**Table 1:** **Kinetic parameters for phenol-degradation of strains isolated from the soil-bioreactor**.

**Isolated strains**	***K*_S_ (μM)**	***K_I_* (μM)**	***V_max_* (μmol min^−1^ g^−1^ of dry cells)**	**No.^a^**	**Accession number**
*Acinetobacter* sp. c1^b^	1.1±0.40	4800±1600	31±1.4	3	AB167183
*Acinetobacter* sp. c26^b^	2.4±1.8	80±30	16±2.4	30	AB167203
*Acinetobacter* sp. c40	11±3.1	530±92	50±4.9	15	AB167208
*Arthrobacter* sp. c106	3.1±0.11	110±12	39±1.2	29	AB167237
*Pseudomonas* sp. LAB-06	2.3±0.31	4800±800	21±0.84	2	AB051693
*Pseudomonas* sp. LAB-08	2.2±0.23	3200±370	30±0.73	4	AB051694
*Pseudomonas* sp. LAB-20	2.8±0.55	1500±290	28±1.5	10	AB051697
*Pseudomonas* sp. LAB-23	2.3±0.23	2700±450	16±0.50	6	AB051699
*Pseudomonas putida* P-2^c^	4.1±1.2	3100±1300	13±1.1	5	AB038136
*Pseudomonas putida* P-5^c^	3.0±0.66	1100±260	48±2.8	12	AB038140
*Pseudomonas putida* P-6^c^	3.9±1.7	460±260	16±2.9	17	AB038141
*Pseudomonas putida* P-8^c^	5.3±1.6	6900±1600	23±1.2	1	AB038142
*Pseudomonas* sp. LAB-26	2.5±0.55	2300±520	33±1.6	7	AB051700
*Ralstonia* sp. P-10^c^	4.4±0.89	580±200	100±20	14	AB016860
*Ralstonia* sp. c5	7.9±1.6	220±60	49±5.3	22	AB167187
*Ralstonia* sp. chemo32	5.1±0.60	470±110	57±3.4	16	LC086859
*Ralstonia* sp. c41	3.2±1.0	200±15	60±10	24	AB167209
*Ralstonia* sp. HAB-01	5.6±0.74	260±46	60±3.5	20	AB051680
*Ralstonia* sp. HAB-02	9.6±1.6	180±37	75±7.5	25	AB051681
*Ralstonia* sp. HAB-11	2.2±0.29	850±120	14±0.45	13	AB051683
*Ralstonia* sp. HAB-18	6.2±0.93	310±70	56±3.7	18	AB051684
Unidentified strain TUT-005	2.9±0.60	1600±350	62±2.8	9	NR^d^
Unidentified strain TUT-006	3.4±0.50	1600±200	130±4.7	8	NR
*Variovorax* sp. c24^b^	8.2±1.2	220±45	93±6.5	21	AB622239
*Variovorax* sp. c52	1.7±0.4	270±20	16±3.1	19	AB167215
*Variovorax* sp. HAB-24^b^	7.6±1.5	180±47	106±12	26	AB051688
*Variovorax* sp. HAB-27	7.1±1.6	120±47	96±15	28	AB051689
*Variovorax* sp. HAB-29	7.4±1.2	150±38	108±11	27	AB051690
*Variovorax* sp. HAB-30^b^	5.8±0.94	200±40	160±12	23	AB051691
*Variovorax* sp. YN07^b^	12±1.4	1200±120	66±2.5	11	AB622227

a *Number links the number shown in Figure 1*.

b *These data were reported in Futamata et al. (2005)*.

c *These data were reported in Futamata et al. (2001a)*.

d *The strain was not registerd in Genbank*.

### Kinetic analyses

Isolated strains or SMEs were grown in a chemostat reactor with BSM medium and phenol as the sole carbon and energy source. The phenol or catechol-oxygenating activity (phenol or catechol-consumption rate) was measured at various substrate concentrations using an oxygen electrode (DO METER TD-51, Toko Chemical Lab. Co., Ltd) after the respiratory oxygen consumption was suppressed by adding potassium cyanide (Watanabe et al., 1996). Kinetic parameters were calculated using the initial phenol-oxygenating velocities at more than 10 different substrate concentrations. The data were fitted to the Michaelis-Menten's equation or the Haldane's equation (Folsom et al., 1990; Watanabe et al., 1998b; Futamata et al., 2005) using JMP statistical visualization software (SAS Institute Inc.). The apparent kinetic constants, *K*_S_ (affinity constant), *K*_I_ (inhibition constant), and *V*_max_ (theoretical maximum activity) were determined using the nonlinear regression method as described previously (Watanabe et al., 1996, 1998a). Following Folsom et al. (1990), the term *K*_S_ was employed instead of *K*_m_ because the activity was measured using intact cells rather than purified enzymes.

### Construction of synthetic microbial ecosystem

Six kinds of SMEs, differing based on their strain composition and flow rate, were constructed with phenol as sole carbon and energy source under chemostat conditions. The SMEI series had 2–4 strains present and a high flow rate while the SMEII series had a lower flow rate and had different combinations of three strains. SMEI-1 consisted of *P. putida* P-8 (Futamata et al., 2001a) and *Variovorax* sp. HAB-24 (Futamata et al., 2001b). SMEI-2 consisted of *P. putida* strains P-8, *Variovorax* sp. HAB-24 and *Acinetobacter* sp. c26 (Futamata et al., 2005). SMEI-3 included *Ralstoni*a sp. c41 in addition to the above-noted 3 strains. It was previously demonstrated that strains P-8 and HAB-24 belong to High-*K*_S_ (Group III) and Low-*K*_S_ (Group I) types according to their nucleotide sequences of the gene cording large subunit of multicomponent phenol hydroxylase (Futamata et al., 2001b). Furthermore, it was shown that the population density of Group III is approximately 10-fold higher than that of Group I. Therefore, initial population density of strain P-8 was set to be 10-fold higher than that of strain HAB-24. The same strains used in SMEI-2 were used in SMEII-1 to investigate the effect of medium flow rate on population dynamics. SMEII-2 consisted of *P. putida* LAB-06 (Futamata et al., 2001b), *Acinetobacter* sp. c26 and *Variovorax* sp. HAB24. SMEII-3 consisted of strain LAB-06, *Ralstonia* sp. chemo32, and *Variovorax* sp. HAB-30 (Futamata et al., 2001b). Prior to inoculation into the chemostat, all strains were precultured at 25°C in BSM medium supplemented with 2 mM phenol as the sole carbon source. Cultures were harvested at the early- or mid-exponential growth phase and then were transferred into 1.5 L of BSM medium containing 0.2 mM of phenol [in a chemostat reactor [(2 L in capacity)]. The initial cell density of each strain was adjusted to approximately 1.0 × 10^5^ cells mL^−1^ by measuring the optical density at 600 nm (OD_600nm_). An OD_600nm_ of 0.1 corresponded to 1.0 × 10^9^ cells mL^−1^ for strains HAB-24 and HAB-30 and to 5.0 × 10^8^ cells mL^−1^ for the other bacteria. After the initially added phenol was almost completely degraded (start-up phase), the SMEI series cultures were supplied continuously with BSM medium containing phenol (1500 mg L^−1^) at a flow rate of 31.5 mL h^−1^, corresponding to a dilution rate (*D*) of 0.5 d^−1^ (31.5 mL h^−1^ × 24 h/1500 mL). The hydraulic residence time (HRT), calculated as 1/*D*, was 2 days. The SMEII series cultures were supplied continuously with BSM medium containing phenol (1500 mg L^−1^) at a flow rate of 10.4 mL h^−1^ (HRT was 6 days). The culture volume was maintained at 1.5 L. The culture was stirred at 150 rpm, and the temperature and pH were maintained at 25°C and 7.0, respectively. Air was filtered through 0.2 μm-pore-size membrane filters (Millipore) and supplied to the culture at 1.5 L min^−1^. The concentration of phenol in the culture was measured using a colorimetric assay with a Phenol Test Wako kit (Wako Pure Chemicals) (Futamata et al., 2001b). The detection limit of this method was around 1.0 μM.

### Monitoring of strains in synthetic microbial ecosystems

The population density of each strain was monitored using real-time qPCR targeting the gene encoding the large subunit of phenol hydroxylase (LmPH). Specific sets of primers were designed by the alignment of various LmPH genes (Supplemental Table 1). A specific PCR-product amplified with each specific primer set was used as a standard DNA fragment in a real-time qPCR analysis. For the monitoring of the *P. putida* P-8, *Variovoras* sp. HAB-24, and *Ralstonia* sp. c41, the PCR profile consisted of preheating at 95°C for 10 min, followed by 40 cycles of denaturation at 95°C for 10 s, annealing at 62°C for 5 s, and extension at 72°C for 15 s. The annealing temperature was set to 56°C and 68°C for the monitoring of strains *Acinetobacter* sp. c26 and *Variovorax* sp. HAB30, respectively. Annealing temperature was set to 58°C for the monitoring of strains *P. putida* LAB-06 and *R*. sp. chemo32. The fluorescence signal was detected at 72°C in each cycle, and a melting curve was obtained by heating the product to 95°C and cooling to 40°C. The reaction was performed using a LightCycler FastStart DNA Master SYBR GREEN I kit (Roche Molecular Biochemicals, Indianapolis, IN, USA) and a LightCycler system (Roche Diagnostics, Mannheim, Germany) according to the manufacturer's instructions. The copy number of each of the amplicons was calculated using the LightCycler software version 3.52.

### Simulation

We performed computer simulations about SMEI-1 (strains P-8 and HAB-24) using the Runge-Kutta method (Saito et al., 1999; Saito, 2002), with important modifications described in the equations below.

$$S\prime =\left(S^{\left(0\right)}-\;S\right)D-\mu_{1}S/(K_{SI}+S)x_{1}/r_{1}-\mu_{2}S/(K_{S2}+S)x_{2}/r_{2}$$

$x_{1}^{\prime }=x_{1}\left\{\mu_{1}S∕\left(K_{SI}+S\right)-D\right\}$ and $x_{2}^{\prime }=x_{2}\left\{\mu_{2}S∕\left(K_{S2}+S\right)-D\right\}$ where *S* is the substrate (phenol in this study) concentration (mg L^−1^), *D* is the dilution rate (h^−1^), μ is the growth rate constant (h^−1^), *x* is the dried cell density (mg L^−1^), *r* is the cell yield (g [cell] g^−1^ [substrate]) and *K*_S_ is the half-saturation constant (μM). Here “1” and “2” mean strains P-8 and HAB-24, respectively.

### Specific growth activity

Since it is known that supernatants of microbial culture can affect the metabolic processes of other microbes (Tanaka et al., 2005; Tashiro et al., 2013; Inaba et al., 2015), the effect of interspecies communication was investigated using a supernatant collected from a pure chemostat culture and was evaluated as specific growth activity. Growth curves were recorded to estimate the physiological changes that occurred after the addition of a supernatant. Each strain was incubated in the BSM medium under the conditions of the chemostat culture supplemented with phenol as the sole carbon source. After the culture was stable, which means that phenol was not detected and OD_600nm_ of the culture reached plateau, the culture was centrifuged at 4°C and 5800 × g. The supernatant was sterilized by filtration through a Steriflip-GP Filter ([pore size is 0.22 μm], Millipore). The supernatant was stored at −20°C after filtration. Frozen supernatant was thawed for each use. We empirically knew that the growth inhibiting activity of supernatant was kept for approximate 6 month at least. The effect of a single supernatant on growth of all strains was always assessed at the same time as a control. Cells precultured in the BSM medium supplemented with 2.0 mM phenol (BSM2.0phe) and 0.3 mL of filter-sterilized supernatant were transferred into 2.7 mL of fresh BSM2.0phe medium. The initial amount of cells was adjusted to an OD_600nm_ of 0.01. As the control condition, 0.3 mL of BSM medium without phenol was added instead of the supernatant. The growth curve was automatically measured using a Bio-photorecorder (TVS062CA, ADVANTEC). Growth parameters, including a lag time (h), growth rate constant (μ [h^−1^]) and amount of growth at a stationary phase (OD_max_), were calculated using the growth curve. Here, we defined the specific growth activity as the surviving activity maintaining the cell density over 1.0 × 10^9^ cells mL^−1^ in a chemostat culture under the condition of a dilution rate. Therefore, 1 unit (U) of specific growth activity was calculated using the following equations: 1 U = 0.021 (h^−1^) × 10^9^ (cells mL^−1^) under the HRT condition of 2 days (in SMEI series) or 1 U = 0.0069 (h^−1^) × 10^9^ (cells mL^−1^) under the HRT condition of 6 days (in SMEII series). As mentioned above, the cell density of *Variovorax* sp. strains HAB-24 and HAB-30 were 1.0 × 10^9^ cells mL^−1^ at an OD_600nm_ of 0.1, whereas the other strains had cell densities of 5.0 × 10^8^ cells mL^−1^ at an OD_600nm_ of 0.1. The OD_max_ was then converted to cell density. Thus, one unit of specific growth activity changes according to the strain and the dilution rate of the chemostat. Units of specific growth activity for the tested strains were calculated according to the following equation:

U=(μ × cell density from ODmax)/(1 U × lag time).

### *J* parameter

*J* parameter was calculated according to the following equation (Hansen and Hubbell, 1980): *J* = (*K*_*s*_ × *D*)/(μ−*D*), where *K*_*S*_ is the half-saturation constant (μM), *D* is the dilution rate (h^−1^), and μ is the growth rate constant (h^−1^). Parameters of *D* in SMEI and SMEII series were 0.021 (h^−1^) and 0.0069 (h^−1^), respectively. Based on the equation, the strain exhibiting a lower *J* parameter should out-grow strains exhibiting higher *J* parameters.

## Results

### Kinetic parameters for phenol of strains

Kinetic parameters for phenol of 30 strains isolated from the soil bioreactor and its inoculum are shown in Table 1 and plotted in Figure 1. Overall, *Pseudomonas* sp. strains, with the exception of *Pseudomonas* sp. P-6, exhibited lower *K*_S_-values (3.0 ± 1.1 μM) and higher *K*_I_-values (3200 ± 1900 μM) than the other strains. The *Ralstonia* sp. strains exhibited middle *K*_S_-values (4.9 ± 1.9 μM) and *K*_I_-values (410 ± 240 μM) with the exception of *Ralstonia* sp. HAB-02. The *Variovorax* sp. strains exhibited higher *K*_S_-values (7.2 ± 0.86 μM) and lower *K*_I_-values (170 ± 40 μM) with the exception of *Variovorax* sp. strains YN07 and chemo52. Almost all of the kinetic parameters for phenol of the individual strains were similar to those from the soil bioreactor as described previously (Haruta et al., 2013). The *K*_S_-values varied within one order of magnitude (from 1 to 10 μM), whereas the *K*_I_-values varied within two orders of magnitude (from 100 to 1000 μM). Furthermore, kinetic parameters (*K*_S_- and *K*_I_-values) of the strains and the soil bioreactor changed one-thirtieth fold decrease in the *K*_I_-values with a three-fold increase in the *K*_S_-value.

**Figure 1:**
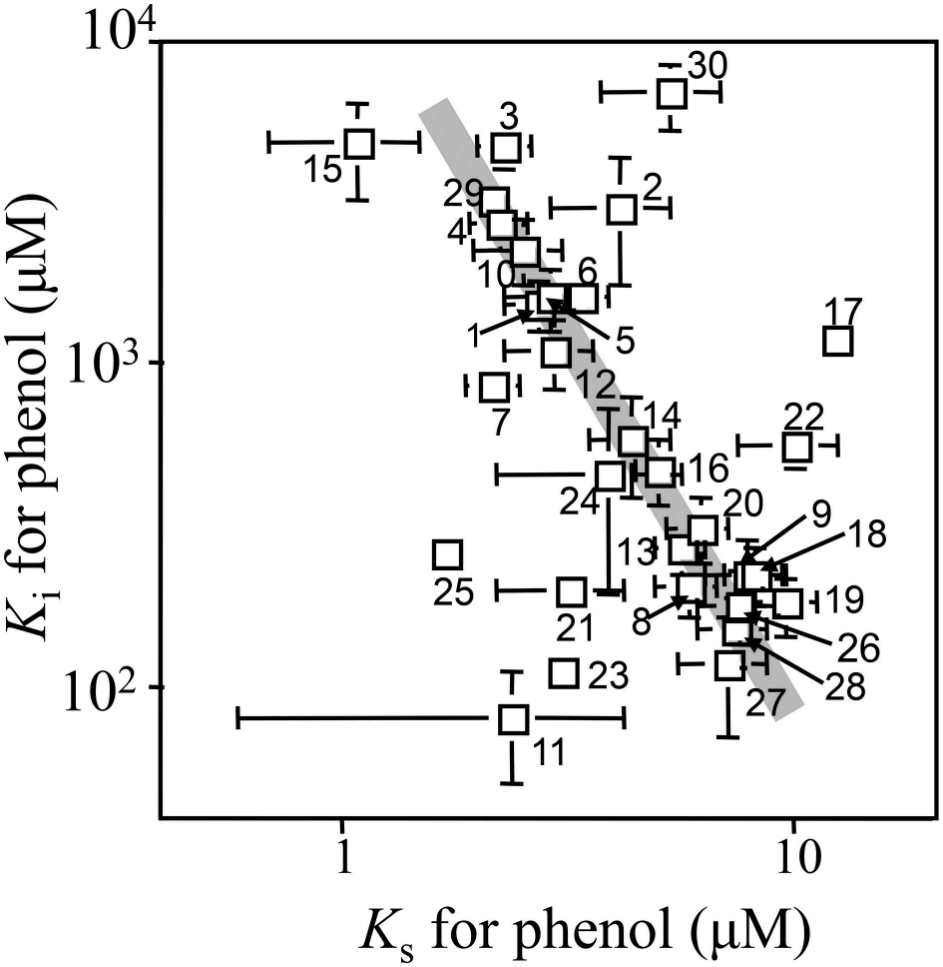
**Kinetic parameters of isolated strains and the soil bioreactor for phenol**. Number indicates the strain shown in Table 1.

### Population dynamics in the mixed chemostat reactor

#### SMEI-1

**Figure 2:**
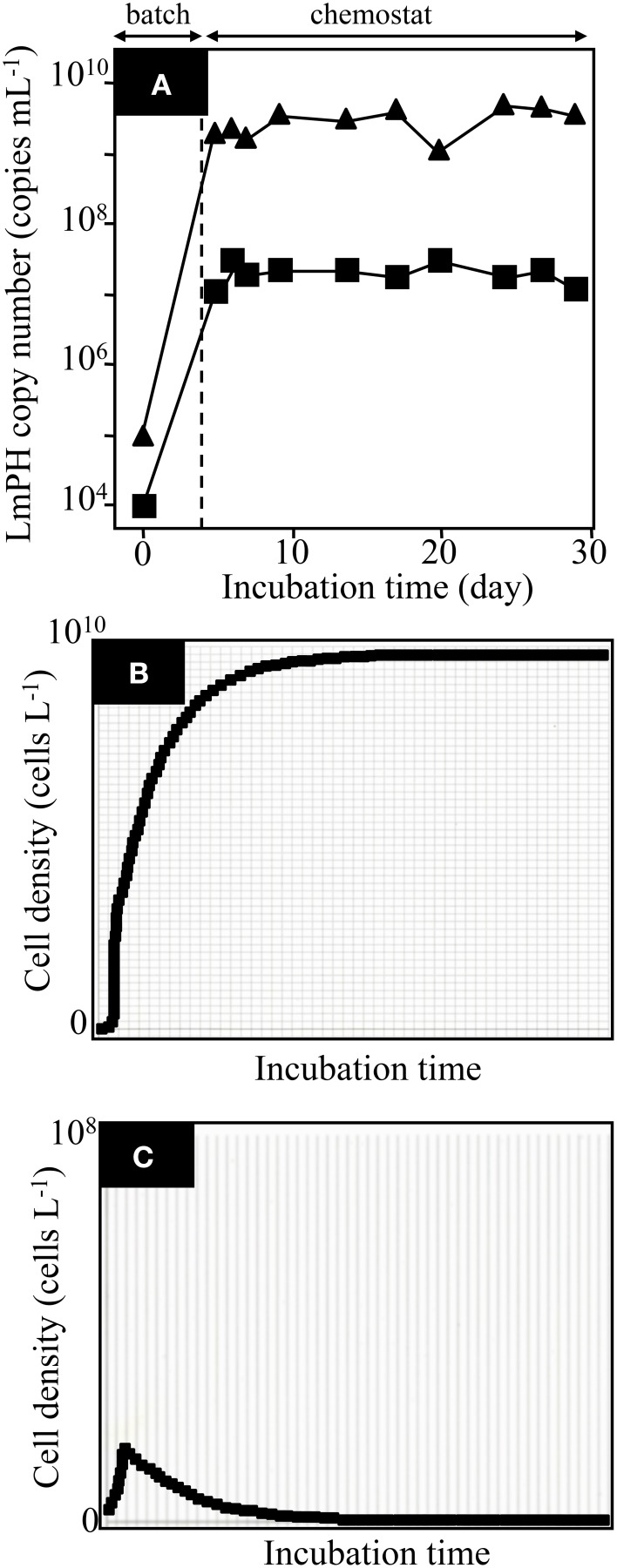
**Population dynamics in the chemostat reactor SMEI-1 run at a flow rate of 31.5 mL h^−1^ (a hydraulic residence time of 2.0 days) and a simulation. (A)** The population densities of the strains in the mixed chemostat culture were monitored using real-time qPCR targeting the gene encoding LmPH. *Psuedomonas putida* P-8 (▴) and *Variovorax* sp. HAB-24 (■). Error bars are smaller than the symbol. **(B)** The result of simulation showing the population of *P*. *putida* P-8. **(C)** The result of simulation showing the population of *Variovorax* sp. HAB-24.

To understand the previous observation that high *K*_S_-type bacteria eventually became dominant in the soil bioreactor, we attempted to reproduce the result in an SME constructed with the isolated strains *P*. *putida* P-8 (No. 1 shown in Figure 1) and *Variovorax* sp. HAB-24 (No. 26 shown in Figure 1). The SME was run at a flow rate of 31.5 mL h^−1^ (an HRT of 2.0 days) and the population densities were monitored by the real-time qPCR technique (Figure 2A). It was previously shown that *Pseudomonas* and *Variovorax* strains were the dominant genera in, respectively, the early and final stages of reactor operation (Futamata et al., 2005). The kinetic parameters of strains P-8 and HAB-24 were similar to those of the soil bioreactor. From these phylogenetic and kinetic data, it was predicted that strain HAB-24 would be the final dominant strain. However, in the SME strain P-8 overcame strain HAB-24 (Figure 2A); the population densities of strains P-8 and HAB-24 were stable at 3.6 ± 1.1 × 10^9^ cells mL^−1^ and 1.3 ± 0.84 × 10^7^ cells mL^−1^, respectively. The result of a simulation showed that only strain P-8 would survive and be maintained at 9.8 × 10^9^ cells mL^−1^ (Figure 2B), whereas strain HAB-24 would be eradicated (Figure 2C).

#### SMEI-2

**Figure 3:**
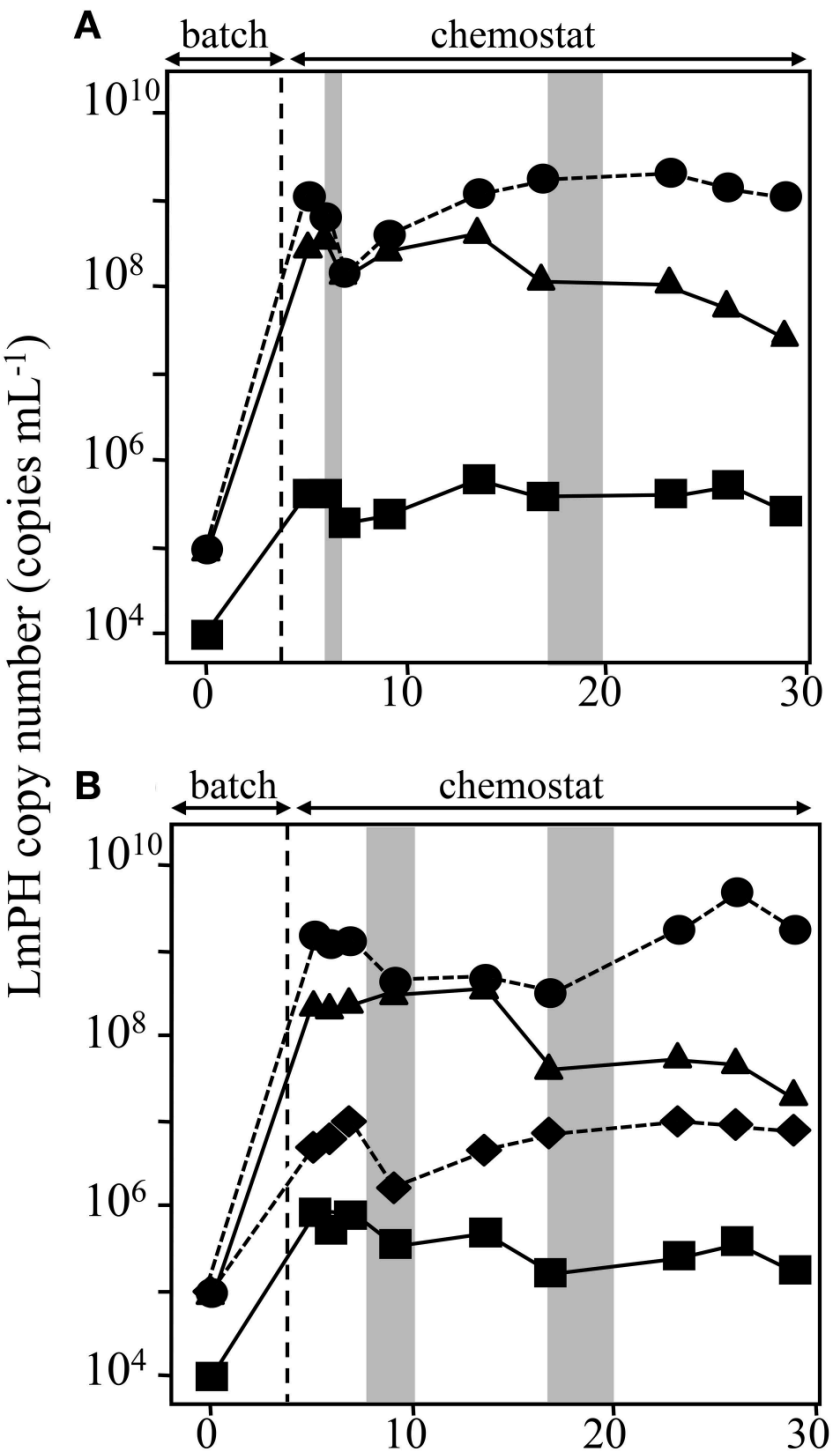
**Population dynamics in the chemostat reactors SMEI-2 and SMEI-3 run at a flow rate of 31.5 mL h^−1^ (a hydraulic residence time of 2.0 days)**. The deduced population densities of the strains in the mixed chemostat culture were monitored using real-time qPCR targeting the gene encoding LmPH. **(A)** Monitoring of the bacterial populations in the SMEI-2 reactor. *Psuedomonas putida* P-8 (▴) *Acinetobacter* sp. c26 (•), and *Variovorax* sp. HAB-24 (■). **(B)** Monitoring of the bacterial populations in the SMEI-3 reactor. *Psuedomonas putida* P-8 (▴) *Acinetobacter* sp. c26 (•) *Ralstonia* sp. c41 (♦), and *Variovorax* sp. HAB-24 (■). Error bars are smaller than the symbol. Gray bar indicates the period when phenol was detected in the reactors.

SMEI-2, which consisted of strains *P. putida* P-8, *Acinetobacter* sp. c26 (No. 30 shown in Figure 1), and *Variovorax* sp. HAB-24, was run at a flow rate of 31.5 mL h^−1^ (an HRT of 2.0 days) and the population densities were monitored using real-time qPCR (Figure 3A). The genera *Pseudomonas, Acinetobacter*, and *Variovorax* corresponded respectively to the first, second, and third dominant genera in the soil bioreactor. According to the affinity dynamics of the soil bioreactor for phenol and catechol, the affinity parameters of strain c26 for phenol and catechol were located at an inflection point from low *K*_S_ for phenol and high *K*_S_ for catechol to high *K*_S_ for phenol and low *K*_S_ for catechol of the soil bioreactor (Supplemental Figure 1). It was thought that a strain located at this inflection point would be needed for strain HAB-24 to become dominant. Strains P-8 and c26, rather than strain HAB-24, grew quickly in the batch mode because of their higher μ-values (Table 2). The population density of strain c26 was maintained at around 1.1 ± 0.073 × 10^9^ cells mL^−1^ after day 5. Although strain P-8 grew well at a similar level to strain c26 under batch mode, the population density of strain P-8 decreased gradually from 6.1 × 10^8^ cells mL^−1^ to 4.7 × 10^7^ cells mL^−1^. On the other hand, the population density of strain HAB-24 remained stable at about 4.11 ± 0.24 × 10^5^ cells mL^−1^ after day 5. Small amounts of phenol were detected in the effluent from the SMEI-2 around day 7 as well as from day 17 to 20 (data not shown).

**Table 2:** **Kinetic and growth parameters on phenol of isolates used in mixed chemostat culture**.

**Strains**	**μ(h^−1^)**	**Maximum OD**	***J* parameter (μM)^a^**	***J* parameter (μM)^b^**
*Acinetobacter* sp. c26	0.63±0.13	0.18±0.046	0.086±0.025	0.027±0.0077
*Ralstonia* sp. chemo32	0.22±0.089	0.27±0.11	0.59±0.17	0.18±0.048
*Ralstonia* sp. c41	0.17±0.029	0.32±0.043	0.46±0.096	0.14±0.026
*Pseudomonas* sp. LAB-06	0.39±0.14	0.23±0.0084	0.41±0.20	0.13±0.060
*Pseudomonas* sp. P-8	0.37±0.054	0.24±0.0093	0.33±0.059	0.10±0.018
*Variovorax* sp. HAB-24	0.11±0.012	0.25±0.020	1.7±0.21	0.49±0.052
*Variovorax* sp. HAB-30	0.13±0.021	0.20±0.084	1.2±0.19	0.33±0.050

a *Dilution rate was 0.021 h^−1^ (HRT = 2 days). It was a chemostat condition of BRI-series*.

b *Dilution rate was 0.0069 h^−1^ (HRT = 6 days). It was a chemostat condition of BRII-series*.

#### SMEI-3

SMEI-3, which consisted of *P*. *putida* P-8, *Acinetobacter* sp. c26, *Ralstonia* sp. c41 (No. 24 shown in Figure 1), and *Variovorax* sp. HAB-24, was run at a flow rate of 31.5 mL h^−1^ (an HRT of 2.0 days) and the population densities were again monitored (Figure 3B). According to the affinity dynamics of the soil bioreactor for phenol and catechol, it was predicted that strain c41 would be an intermediate between strains P-8, HAB-24, and c26 (Supplemental Figure 1). The amount of growth of the four strains corresponded to their μ-values under batch conditions. Their population densities were roughly maintained under chemostat conditions of this experiment; the population densities of strains c26, P-8, c41, and HAB-24 were 1.3 ± 0.21 × 10^9^ cells mL^−1^, 2.3 ± 0.26 × 10^8^ cells mL^−1^, 7.6 ± 0.81 × 10^6^ cells mL^−1^, and 2.9 ± 1.71 × 10^5^ cells mL^−1^, respectively. Again, small amounts of phenol were detected in the effluent from the SMEI-3 on days 7–10 and days 16–20 (data not shown).

#### SMEII-1

**Figure 4:**
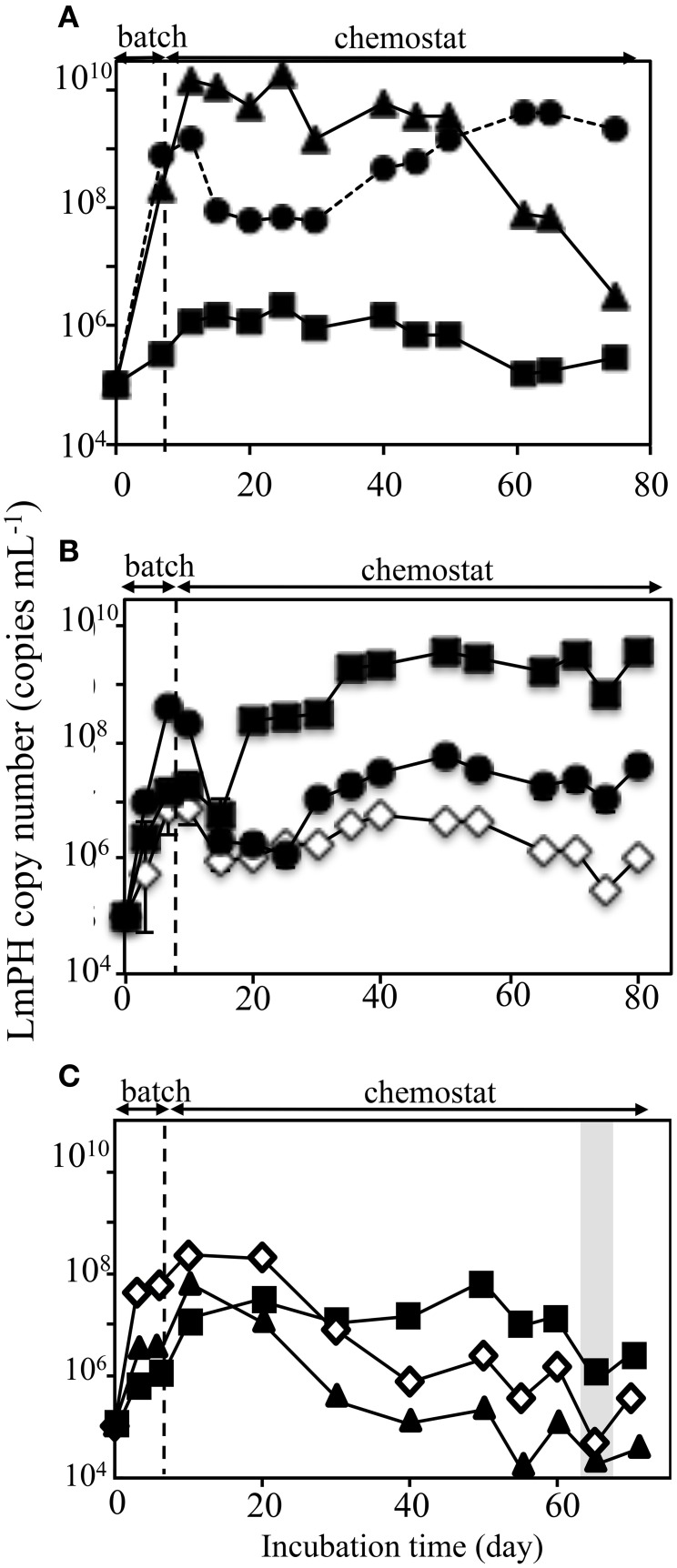
**Population dynamics in the chemostat reactors SMEII series run at a flow rate of 10.4 mL h^−1^ (a hydraulic residence time of 6.0 days)**. The deduced population densities of the strains in the mixed chemostat culture were monitored using real-time qPCR targeting the gene encoding LmPH. **(A)** Monitoring of the bacterial populations used in the SMEII-1 reactor. *Psuedomonas putida* P-8 (▴) *Acinetobacter* sp. c26 (•), and *Variovorax* sp. HAB-24 (■). **(B)** Monitoring of the bacterial populations used in the SMEII-2 reactor. *Psuedomonas* sp. LAB-06 (♢), *Acinetobacter* sp. c26 (•), and *Variovorax* sp. HAB-24 (■). **(C)** Monitoring of the bacterial populations used in the SMEII-3 reactor. *Psuedomonas* sp. LAB-06 (♢), *Acinetobacter* sp. chemo32 (▴), and *Variovorax* sp. HAB-24 (■). The gray bar indicates the period when phenol was detected in the reactors. The error bars indicate standard deviations and some bars were hided with symbols.

SMEII-1 consisted of *P*. *putida* P-8, *Acinetobacter* sp. c26, and *Variovorax* sp. HAB-24, which were the same strains as used in SMEI-2). The purpose of SMEII-1 was to investigate the effect of flow rate on population dynamics. Here the flow rate was decreased from 31.5 mL h^−1^ (HRT = 2.0 days) to 10.4 mL h^−1^ (HRT = 6.0 days). The population densities were monitored using real-time QPCR with specific sets of primers (Figure 4A). Strains P-8 and c26 grew faster than strain HAB-24 in the batch mode, as was the case in SMEI-2. The population density of strain P-8 was stable at 6.2 ± 0.78 × 10^9^ cells mL^−1^ from day 10 to day 48, after which the population density decreased below 1.0 × 10^7^ cells mL^−1^ by the end of the experiment. Conversely, the population density of strain c26 was stable at 8.2 ± 0.16 × 10^7^ cells mL^−1^ from day 15 to day 30, after which the population density increased to approximately 4.2 ± 0.32 × 10^9^ cells mL^−1^ by the end of the experiment. The population density of strain HAB-24 was stable at around 6.2 ± 0.76 × 10^5^ cells mL^−1^ after day 5. The population dynamics of SMEII-1 were similar to that of SMEI-2, showing that the final dominant bacterium was *Acinetobacter* sp. c26. Phenol was never detected in the effluent during this experiment. Therefore, all subsequent SMEII series chemostats were run with a flow rate at 10.4 mL h^−1^ (HRT = 6.0 days).

#### SMEII-2

SMEII-2 was constructed with *Pseudomonas* sp. LAB-06 (No. 2 shown in Figure 1), *Acinetobacter* sp. c26 and *Variovorax* sp. HAB-24, and run with a flow rate of 10.4 mL h^−1^ (an HRT of 6.0 days) (Figure 4B). Strain c26, but not strains LAB-06 and HAB-24, grew quickly in the batch mode because of higher μ-value (Table 2). Strain c26 remained dominant until day 10. On day 15, the population densities of the three strains were all close to 2.8 ± 2.5 × 10^6^ cells mL^−1^, after which the population density of HAB-24 increased and was stable at 2.5 ± 1.1 × 10^9^ cells mL^−1^ after day 35. Conversely, the population density of LAB-06 and c26 were 2.6 ± 1.8 × 10^6^ cells mL^−1^ and 2.7 ± 1.6 × 10^7^ cells mL^−1^ after day 30, respectively. Bacterial community succession was observed, resulting in the dominance of HAB-24.

#### SMEII-3

SMEII-3 was constructed with *Pseudomonas* sp. LAB-06 (No. 2 shown in Figure 1), *Ralstonia* sp. chemo32 (No. 16 shown in Figure 1), and *Variovorax* sp. HAB-30 (No. 23 shown in Figure 1) and run at a flow rate of 10.4 mL h^−1^ (an HRT of 6.0 days) (Figure 4C). Strains LAB-06 and chemo32 grew faster than strain HAB-30 in the batch mode because of their higher μ-values (Table 2). Strain LAB-06 became dominant at a cell density of 1.6 ± 0.89 × 10^8^ cells mL^−1^ until day 20. The population densities of strains LAB-06 and chemo32 decreased to 9.1 ± 8.9 × 10^5^ cells mL^−1^ and 8.6 ± 8.2 × 10^4^ cells mL^−1^, respectively, after day 40. Conversely, the population density of HAB-30 remained stable at around 1.3 ± 1.3 × 10^7^ cells mL^−1^ from days 10 to 60. Thus, SMEII-3 exhibited a bacterial community succession, where strain HAB-30 became dominant. Small amounts of phenol were detected in the effluent from the SMEII on days 65–70 (data not shown).

### Interspecies interactions

#### Strains used in the SMEI series SMEs

Interactions among the four strains used in the SMEI series SEMs, *P*. *putida* P-8, *Variovorax* sp. HAB-24, *Acinetobacter* sp. c26, and, *Ralstonia* sp. c41, were investigated by measuring their specific growth activities (Table 3). The supernatant of strain P-8 culture enhanced the growth activities of strains P-8 and c26 to approximately 140 and 180%, respectively but repressed those of strains c41 and HAB-24 to approximately 6.5 and 7.4%, respectively. The supernatant of strain c26 did not affect the growth activities of strains P-8 and c26, but repressed those of strains c41 and HAB-24 to approximately 25 and 38%, respectively. The supernatant of strain c41 enhanced the growth activities of strains P-8 and c26 to approximately 120% but did not affect the growth activities of strains c41 and HAB-24. The supernatant of strain HAB-24 enhanced the growth activities of strains c26 and HAB-24 to approximately 160 and 150%, respectively, but repressed those of strains P-8 and c41 to approximately 40 and 11%, respectively.

**Table 3:** **Relative specific growth among strains used in SMEI-series and SMEII-1^a^**.

**Strains**	**Supernatant of strains**			
	**P-8**	**c26**	**chemo41**	**HAB-24**
*Pseudomonas* sp. P-8	140±35	105±11	120±16	40±5.0
*Acinetobacter* sp. c26	180±90	100±3.0	120±58	160±90
*Ralstonia* sp. c41	6.5±0.5	25±9.3	97±39	1±9.1
*Variovorax* sp. HAB-24	7.4±1.3	38±8.6	90±12	153±8.9

a *The specific growth activity without supernatant (control condition) was calculated as 100%*.

#### Strains used in the SMEII series SMEs

Interactions among the three strains used in SMEII-2 (Table 4) and SMEII-3 (Table 5) were investigated by measuring their specific growth activities. The supernatant of strain LAB-06 did not affect its own growth activity but it repressed that of strain c26 to approximately 71% and enhanced that of strain HAB-24 to approximately 180% (Table 4). Supernatant of strain c26 did not affect the growth activity of strain LAB-06 and itself but repressed that of strain HAB-24 to approximately 38%. Supernatant of strain HAB-24 repressed the growth activity of strain LAB-06 to approximately 40% but enhanced those of strains c26 and HAB-24 to approximately 160 and 153%, respectively. The supernatant of strain LAB-06 repressed those of strains chemo32 and HAB-30 to approximately 75 and 22%, respectively (Table 5). The supernatant of strain chemo32 did not affect the growth activities of strains LAB-06 and chemo32 but enhanced that of strain HAB-30 to approximately 160%. The supernatant of strain HAB-30 enhanced its own growth activity to approximately 140% but repressed those of strains LAB-06 and chemo32 to approximately 87 and 48%, respectively. The mixed supernatant of strains chemo32 and HAB-30 did not affect the growth activity of LAB-06. Similarly, the mixed supernatant of strains LAB-06 and chemo32 did not affect the growth activity of HAB-30. The mixed supernatant of strain LAB-06 and HAB-30 repressed the growth activity of chemo32 to approximately 85%.

**Table 4:** **Relative specific growth among strains used in SMEII-2^a^**.

**Strains**	**Supernatant of strains**		
	**LAB-06**	**c26**	**HAB-24**
*Pseudomonas* sp. LAB-06	93±7.3	105±11	77±8.4
*Acinetobacter* sp. c26	71±6.8	100±3.0	160±90
*Variovorax* sp. HAB-24	180±90	38±8.6	153±8.9

a *Specific growth activity without supernatant was calculated as 100% (control condition)*.

**Table 5:** **Relative specific growth among strains used in SMEII-3^a^**.

**Strains**	**Supernatant of strains**			
	**LAB-06**	**chemo32**	**HAB-30**	**Mixed supernatant**
*Pseudomonas* sp. LAB-06	93±7.3	103±41	87±12	103±8.5^b^
*Ralstonia* sp. chemo32	75±6.3	100±50	48±17	85±16^c^
*Variovorax* sp. HAB-30	22±6.4	160±35	140±33	105±4.8^d^

a *Specific growth activity without supernatant (control condition) was calculated as 100%*.

b *Supernatants obtained from strain c32 and HAB-30 were used*.

c *Supernatants obtained from strain LAB-06 and HAB-30 were used*.

d *Supernatants obtained from strain LAB-06 and c32 were used*.

### Dynamics of kinetics parameters in the SMEII-3 SME

**Figure 5:**
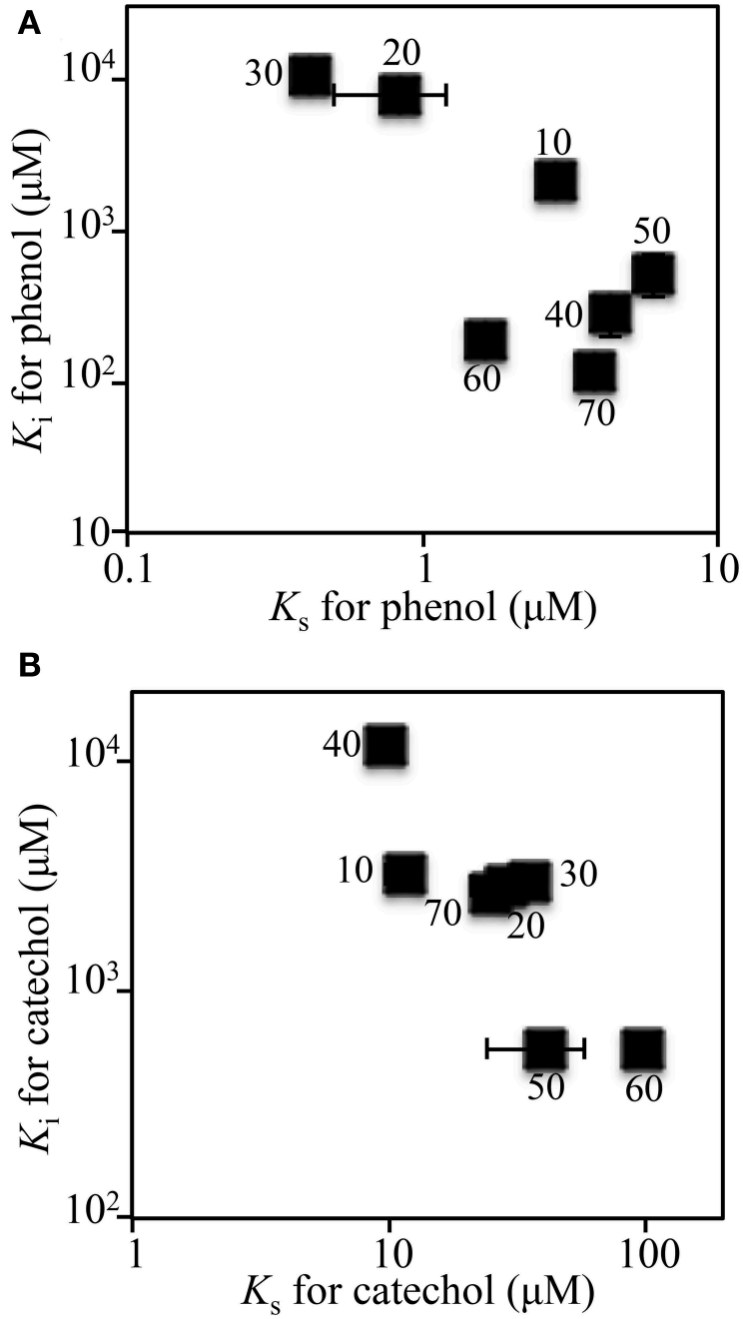
**Kinetic parameters for phenol (A) and catechol (B) of the SMEII-3**. Number indicates the sampling date. The error bars indicate standard deviations and some bars were hided with symbols.

The kinetic parameters (*K*_S_- and *K*_I_-values) of the SMEII-3 SME for phenol and catechol were monitored (Figure 5). The *K*_S_- and *K*_I_-values for phenol shifted from 2.6 ± 0.17 μM and 2060 ± 200 μM at day 10 to 0.42 ± 0.037 μM and 1060 ± 28 μM at day 30, which indicated that the affinity for phenol of the SMEII-3 reactor increased. However, these kinetic parameters changed dramatically and exhibited a dynamic equilibrium at higher *K*_S_-values (4.0 ± 1.8 μM) and lower *K*_I_-values (275 ± 180 μM) from days 40 to 70. The *K*_S_-value for catechol shifted more dynamically than that for phenol (Figure 5B). The *K*_S_- and *K*_I_-values for catechol were stable at 25 ± 13 μM and 3000 ± 170 μM, respectively, from days 10 to 30. However, these kinetic parameters fluctuated dynamically from 9.9 ± 0.56 μM and 11500 ± 860 μM at day 40 to 100 ± 6.2 μM and 540 ± 47 μM at day 60 and then returned to their initial values at day 70.

## Discussion

In a soil bioreactor fed with phenol we previously observed a unique phenomenon where the kinetic parameters shifted toward higher *K*_S_ and lower *K*_I_-values (Futamata et al., 2005; Haruta et al., 2013). In the present study, to understand this unique phenomenon, SMEs were constructed using isolated strains exhibiting different phenotypic features. By observing changes in population densities we have developed a better understanding of population dynamics in SMEs and soil bioreactors.

Kinetic parameters have been thought to be one of the most important determinants for predicting the competitiveness of strains grown on a single substrate (Watanabe et al., 1996, 1998a). Since the *J* parameter is calculated using not only the *K*_S_-value (affinity) but also μ (growth rate constant) and *D* (dilution rate), it is especially useful for predicting what strain will become dominant in a chemostat culture (Hansen and Hubbell, 1980). Additionally, cell-to-cell interactions are important for understanding the microbial ecosystem (Watts and Strogatz, 1998; Flagan et al., 2003; Kato et al., 2005; Narisawa et al., 2008). To the best of our knowledge this study presents the first attempt to use both the *J* parameter and interspecies interaction information simultaneously for understanding the dynamics of microbial community in a complex system.

It is reported that *Variovorax* strains are capable of utilizing or producing acyl-homoserine lactones which are quorum signaling molecules in many species of the class *Proteobacteria* (Leadbetter and Greenberg, 2000; d'Angelo-Picard et al., 2005; Yang et al., 2006; Satola et al., 2013). We hypothesized that the *Variovorax* spp. became dominant in the soil bioreactor through the use of quorum signaling. The SMEI series and SMEII-1 were expected to reproduce the unique phenomenon of the soil bioreactor, however, *Variovorax* sp. strain HAB-24 exhibiting highest *K*_S_-value among strains used in SMEs did not become dominant (Figures 2, 3, 4A). Therefore, it was demonstrated that either the *J* parameter or interspecies interactions data were capable of predicting the experimentally observed population dynamics and it was natural that *Acinetobacter* sp. strain c26 became dominant.

It remained unknown whether the *J* parameter or interspecies interactions were more important for predicting population dynamics in a chemostat culture. Therefore, SMEII-2 was constructed with *Acinetobacter* sp. strain c26, *Variovorax* sp. strain HAB-24, and *Pseudomonas* sp. strain LAB-06 instead of *P. putida* strain P-8. Based on their *J* parameters (Table 2), *Acinetobacter* sp. strain c26 was predicted to become dominant. However, from these interspecies interactions (**Table 4**), it was predicted that *Variovorax* sp. strain HAB-24 would become dominant as strain LAB-06 would repress strain c26 and enhance strain HAB-24. In actuality the final dominant organism was strain HAB-24 not strain c26 (Figure 4B), suggesting that the interspecies interactions did indeed play a more important role in bacterial community dynamics than the *J* parameter. To confirm this possibility, SMEII-3 was constructed with *Pseudomonas* sp. strain LAB-06, *Ralstonia* sp. strain chemo32, and *Variovorax* sp. strain HAB-30. Although strain LAB-06 exhibited the lowest *J* parameter (indicating it should be the most competitive) among these three strains (Table 2), the final dominant was strain HAB-30. While interspecies interaction data (Table 5) indicated that strain LAB-06 inhibited HAB-30, the addition of chemo32 was also expected to rescue this inhibition. Thus, the complexity of this three-way interaction resulted in strain HAB-30 becoming dominant after community succession and fluctuation of kinetic parameters (Figure 5). Importantly it was also demonstrated that this SME reproduced the unique phenomenon previously observed in the soil bioreactor (Futamata et al., 2005; Haruta et al., 2013).

Since it is recognized that interactive networks develop among diverse microbes in natural ecosystems (Gilbert et al., 2012), it was expected that understanding these complex interactions would be important for understanding how microbial ecosystems develop. Striking a balance between the enhancing and repressing relationships was considered to be essential for maintaining the stable coexistence of the five bacterial strains in a cellulose-degrading community (Kato et al., 2005). While it is possible to qualitatively understand such interspecies interactions, it remains difficult to quantify their outcome. As a physiological process with defined, quantifiable output, cell growth was expected to be an ideal indicator for understanding the effects of interspecies interactions. Here, using relative growth in the presence of supernatants of other strains, we have demonstrated that the specific growth activity was indeed useful for understanding the dynamics of microbial ecosystems and for predicting the dominant species in a mixed, SME.

In this study we characterized both kinetic, physiological traits of multiple phenol-degrading strains as well as their binary interactions with each other. Using this quantitative method for evaluating interspecies interactions we have developed a method for predicting microbial community dynamics and demonstrated that the complex interactions between species are a more significant determinant for microbial community dynamics than the *J* parameter is for predicting which species will become dominant. Interestingly, although bacterial community succession was observed, all strains still co-existed in all SMEs and none were eradicated, suggesting that these strains shared a role in phenol degradation. Further work still needs to be done as it was thought that these strains shared a role in degradation of phenol by changing metabolic process. A novel biomathematical theory is also required to fully understand dynamic microbial ecosystems. Research subjects that attract attention with respect to microbial community dynamics are relevant to the identification of growth-repressing compounds, mechanisms of interspecies interactions and comparison of metabolism under the conditions of pure and complex cultures.

### Conflict of interest statement

The authors declare that the research was conducted in the absence of any commercial or financial relationships that could be construed as a potential conflict of interest.
