# Supplementary material for: Interspecies interactions are an integral determinant of microbial community dynamics
Source: Front Microbiol. 2015 Oct 20;6:1148. doi: 10.3389/fmicb.2015.01148 (PMC4611161; doi:10.3389/fmicb.2015.01148)
Supplement: Supplementary file 1 [file Presentation1.PDF]

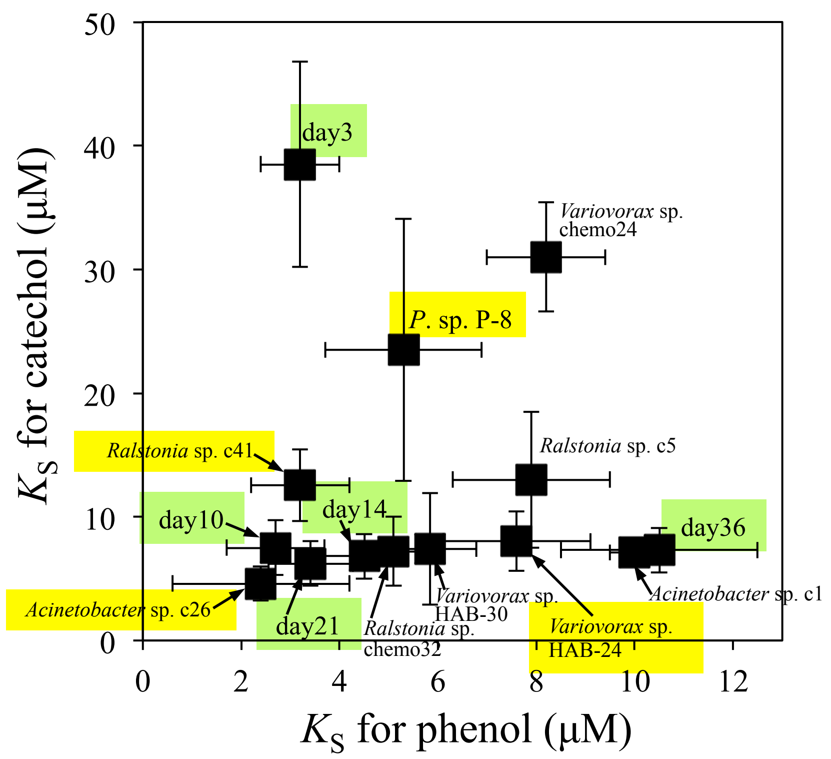

This figure shows the affinity for phenol and catechol of the soil-bioreactor and isolated strains. These days colored in green indicated the sampling date from the soil-bioreactor. The name of isolates used in the BRI-reactors were colored in yellow.

Supplemental Figure 1 Fatma et al.
