# Supplementary material for: Interspecies interactions are an integral determinant of microbial community dynamics
Source: Front Microbiol. 2015 Oct 20;6:1148. doi: 10.3389/fmicb.2015.01148 (PMC4611161; doi:10.3389/fmicb.2015.01148)
Supplement: Supplementary file 2 [file Table1.PDF]

Supplemental Table 1. Nucleotide sequences of specific primers for monitoring LmPH gene

Strain	Specific primers	Nucleotide sequence
Acinetobacter sp. c26	pheAc1f	5'-AGGCTATGCCCATGTTGGACGTCA-3'
	pheAc1r	5'-TTATCTTCGTGCTGTTCCAGT-3'
Ralstonia sp. c32	chemo32f	5'-CCACTTGGGCAACTATCG-3'
	chemo32r	5'-TTTGGCGCGACCAAGATG-3'
Ralstonia sp. c41	chemo041f	5'-TGGCCCTCGCAGGCTACATG-3'
	chemo041r	5'-GGTGAGTCCGCCAAACCG-3'
Pseudomonas sp. LAB-06	LAB06f	5'-CGTTATGTCAACGCACTG-3'
	LAB06r	5'-GCGCATCTTCGAAGAATG-3'
Psuedomonas sp. P-8	P-8f	5'-TGTCCGGCGTGACGCCA-3'
	P-8r	5'-TCGAAGAACGACTTGGGCACT-3'
Variovorax sp. HAB-24	pheVf	5'-CGGCTATGCCATGGCCGGCGCTTC-3'
	pheVr	5'-TTGTCCGGGTCCTGCTCGAGG-3'
Variovorax sp. HAB-30	HAB30f	5'-GCCTGCCAGATGCAGTCG-3'
	HAB30r	5'-AAGGGCATGAACAGCAGG-3'
